# Vestibular Neuritis Associated With COVID-19 Infection: A Case Report and Literature Review on the Time Course and Recovery

**DOI:** 10.7759/cureus.84123

**Published:** 2025-05-14

**Authors:** Saya Masutani, Toru Miwa, Kishiko Sunami

**Affiliations:** 1 Otolaryngology, Osaka Metropolitan University, Osaka, JPN; 2 Otolaryngology, Teikyo University Hospital, Mizonokuchi, Kawasaki, JPN

**Keywords:** covid-19, posturography, vemp, vestibular neuritis, vhit

## Abstract

The pathophysiology of coronavirus disease 2019 (COVID-19) remains elusive, although it has been associated with symptoms such as dizziness and vertigo. Here, we report a case of vestibular neuritis following COVID-19 in a 57-year-old man who presented with rotational vertigo and dizziness exacerbated by neck rotation. The video head impulse test (vHIT) and ocular vestibular evoked myogenic potential (oVEMP) initially indicated right superior vestibular neuritis. Over a seven-month follow-up, improvements in vHIT and oVEMP were consistent with typical vestibular neuritis recovery; however, persistent postural instability and prolonged orthostatic hypotension suggested additional autonomic nervous system dysfunction. This case highlights the potential long-term vestibular impact of COVID-19.

## Introduction

The World Health Organization declared coronavirus disease 2019 (COVID-19) a pandemic on March 11, 2020. Among individuals with COVID-19, dizziness has been reported in 0.03%-30% of patients during the acute phase and in approximately 10% during the follow-up period [1]. Vertigo is less common, affecting 0.004%-12% of patients [2].

These symptoms may result from the direct effects of COVID-19 on the cochlea and peripheral vestibular organs, or from nerve stimulation via neuropathy or virus-mediated immune responses. This is supported by findings of severe acute respiratory syndrome coronavirus 2 (SARS-CoV-2) viral proteins in cranial nerves and cells from the lower brainstem. Potential viral invasion routes include the blood vessels, nerves, and meninges [3].

In patients experiencing COVID-19-related dizziness, vestibular function examinations have produced different findings. Subjective assessments, such as the dizziness handicap inventory (DHI), have demonstrated a decline in self-reported scores. Objective tests, including ocular vestibular evoked myogenic potential (oVEMP) and cervical vestibular evoked myogenic potential (cVEMP) for otolith function, and the video head impulse test (vHIT) for lateral semicircular canal (LSC) function, have demonstrated decline in some studies (e.g., one study of nine adults [4]). The vestibular system comprises the otolith organs and the semicircular canals. Vertigo resulting from a viral infection may be attributed to damage within this system, and identifying the specific site of dysfunction is important for diagnosis and treatment. cVEMP and oVEMP assess otolith organ function in response to acoustic stimulation, whereas vHIT evaluates semicircular canal function by measuring the vestibulo-ocular reflex (VOR) elicited by rapid, passive head rotations. In contrast, other studies have not reported a decline in vestibular function [3]. Additionally, possible brainstem or central lesion involvement has been suggested based on standing tests, imaging, and autopsy reports [5]. However, a clear consensus in the field has yet to be reached. Balance-function tests comparing patients who had COVID-19 with and without dizziness during recovery (22-114 days after onset) showed that cVEMP, oVEMP, and computerized dynamic posturography findings were considerably worse in patients with dizziness. This cross-sectional case-control study compared vestibulocochlear function among COVID-19 outpatients, inpatients, and matched controls (n = 35 each). Taken together, these findings suggest that COVID-19-related vestibular disorders may contribute to long-term sequelae [6]. However, no consistent correlation with disease severity was observed. The findings remain associative and hypothesis-generating [6]. Meanwhile, the inconsistent outcomes imply that the dizziness and vertigo linked to COVID-19 may arise from multiple mechanisms. Thus, long-term follow-up of cases with clearly localized lesion sites is necessary to better understand COVID-19-induced vestibular symptoms.

Vestibular neuritis has been documented in nine COVID-19-related cases of dizziness and vertigo [7-15]. However, specific results of balance-function tests after COVID-19-induced vestibular neuritis, aside from caloric test results, are limited [9]. In addition, evidence regarding the recovery of vestibular function during convalescence remains scarce. Here, we report a case of vestibular neuritis following COVID-19.

## Case presentation

A 57-year-old man experienced sudden-onset rotational vertigo and fever for 10 hours while at work. Emergency computed tomography (CT) and magnetic resonance imaging (MRI) ruled out central pathology. A pharyngeal swab confirmed SARS-CoV-2 infection (antigen > 5,000 pg/mL). Although the vertigo resolved, residual dizziness, exacerbated by neck rotation, persisted. Two months later, he presented to our hospital. His only significant history was a gastric ulcer. Enzyme immunoassay revealed elevated immunoglobulin G levels for herpes simplex virus-1 (50.8 U/mL) and varicella zoster virus (VZV; 15.4 U/mL).

Initial findings

**Figure 1:**
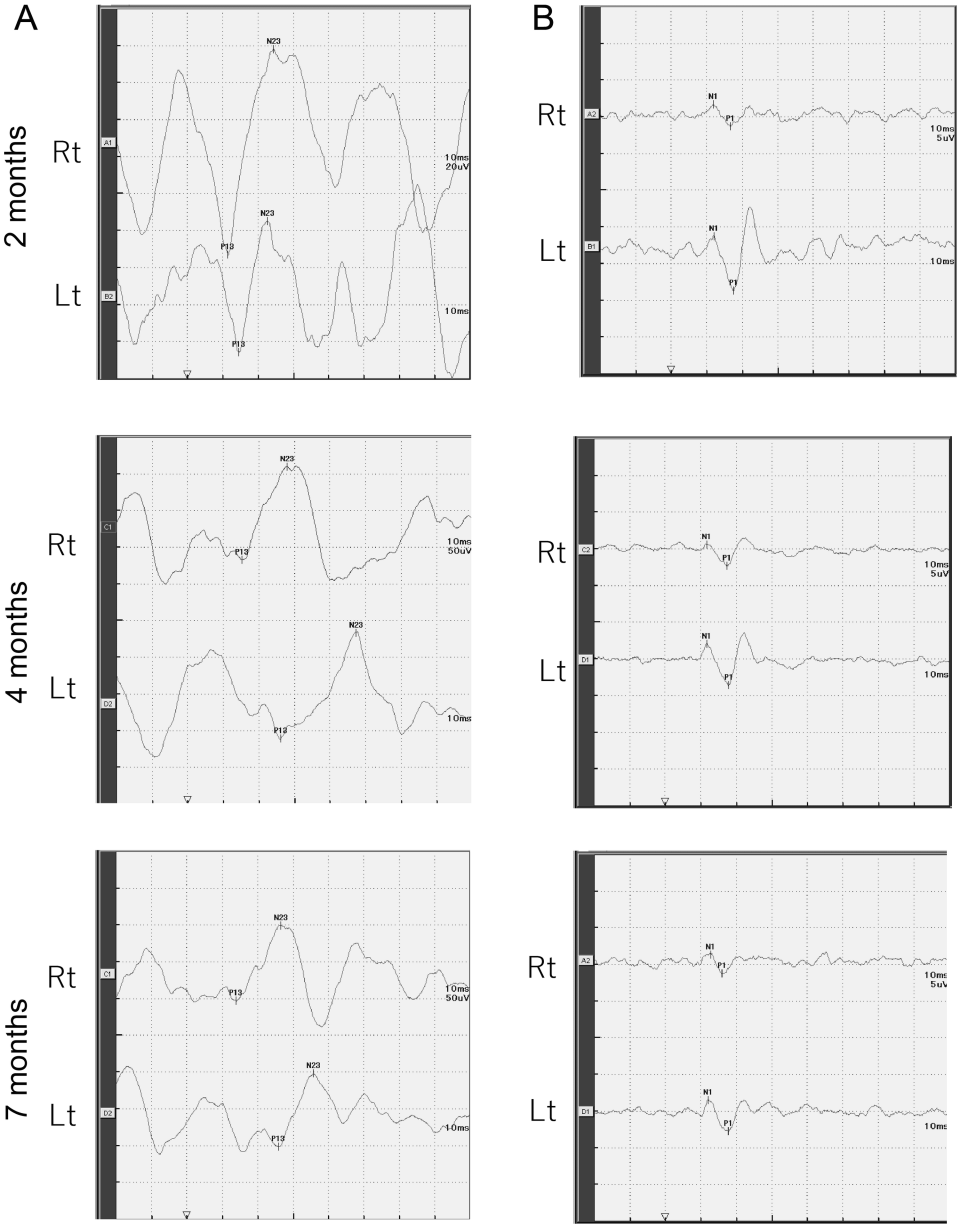
Course of cVEMP and oVEMP Temporal changes in the waveform of cVEMP (A) and oVEMP (B). Rt: right, Lt: left, cVEMP: cervical vestibular evoked myogenic potential, oVEMP: ocular vestibular evoked myogenic potential.

Neurological examination showed spontaneous left-beating nystagmus. Pure-tone audiometry was normal, but caloric testing showed absent responses from the right ear, significant canal paresis (CP), and a residual left-sided response (3.4°/s). vHIT revealed reduced VOR gain (0.76) in the right LSC (Table 1). Owing to limited informed consent, additional vHIT testing could not be performed. cVEMP was normal, whereas oVEMP was reduced on the right side (Figures 1A, 1B). These findings indicated right superior vestibular neuritis.

**Table 1:** Course of the equilibrium test results and symptoms rt: right, lt: left, MVS: maximum velocity speed, vHIT: video head impulse test, LSC: lateral semicircular canal, cVEMP: cervical vestibular evoked myogenic potential, AR%: asymmetric ratio %, oVEMP: ocular vestibular evoked myogenic potential, FT: Foulage test, HUT: head-up tilt, INOH: instantaneous orthostatic hypotension, OH: orthostatic hypotension.

Test/symptoms	Subtype	Two months	Four months	Seven months	Reference (normal)
Symptoms		Dizziness, exacerbated by neck rotation	Dizziness, exacerbated by standing	Light dizziness, exacerbated by gait	
Caloric test	rt/lt MVS (20 ℃)	0/3.4	-	-	>10
vHIT	rt LSC	0.76	0.74	1.96	>0.80
cVEMP	AR%	22.2	6.39	1.14	<33
	Amplitude (μV) rt/lt	111/70	126/144	102/99	50-200
	Latency (ms) p13-n23 rt/lt	11.4-24.4/14.6-22.6	15.3-27.9/26.1-47.3	13.9-26.6 /25.8-35.7	p13:13-15 n23:22-25
oVEMP	AR%	42.7	23.5	31.3	<33
	Amplitude (μV) rt/lt	2.98/7.43	2.63/4.25	2.94/5.63	5-15
	Latency (ms) n10-p15 rt/lt	11.9-16.6/12.1-17.5	12.7-15.9/12.0-17.8	11.8-17.3/11.7-17.9	n10:9-11 p15:14-17
Posturography	Closed eyes velocity with rubber load (vestibular-dependent)	5.94	10.59	8.95	5-10
	Romberg ratio with rubber load (visual-dependent)	1.84	2.09	2.66	1.2-2.5
	Closed eyes with rubber load ratio (somatosensory-dependent)	1.75	1.93	3.27	1.5-3.0
Foulage test	FT value open/closed eyes	5.33/9.13	4.93/6.55	7.05/7.61	Not yet released
	Variance of steps open/closed eyes	2.50/6.52	2.28/2.27	4.45/3.08	Not yet released
	Θ value open/closed eyes	-0.32/9.28	3.00/-3.00	-2.00/-6.00	Not yet released
HUT		INOH	Normal	Delayed OH	Blood pressure and heart rate fluctuate less

**Figure 2:**
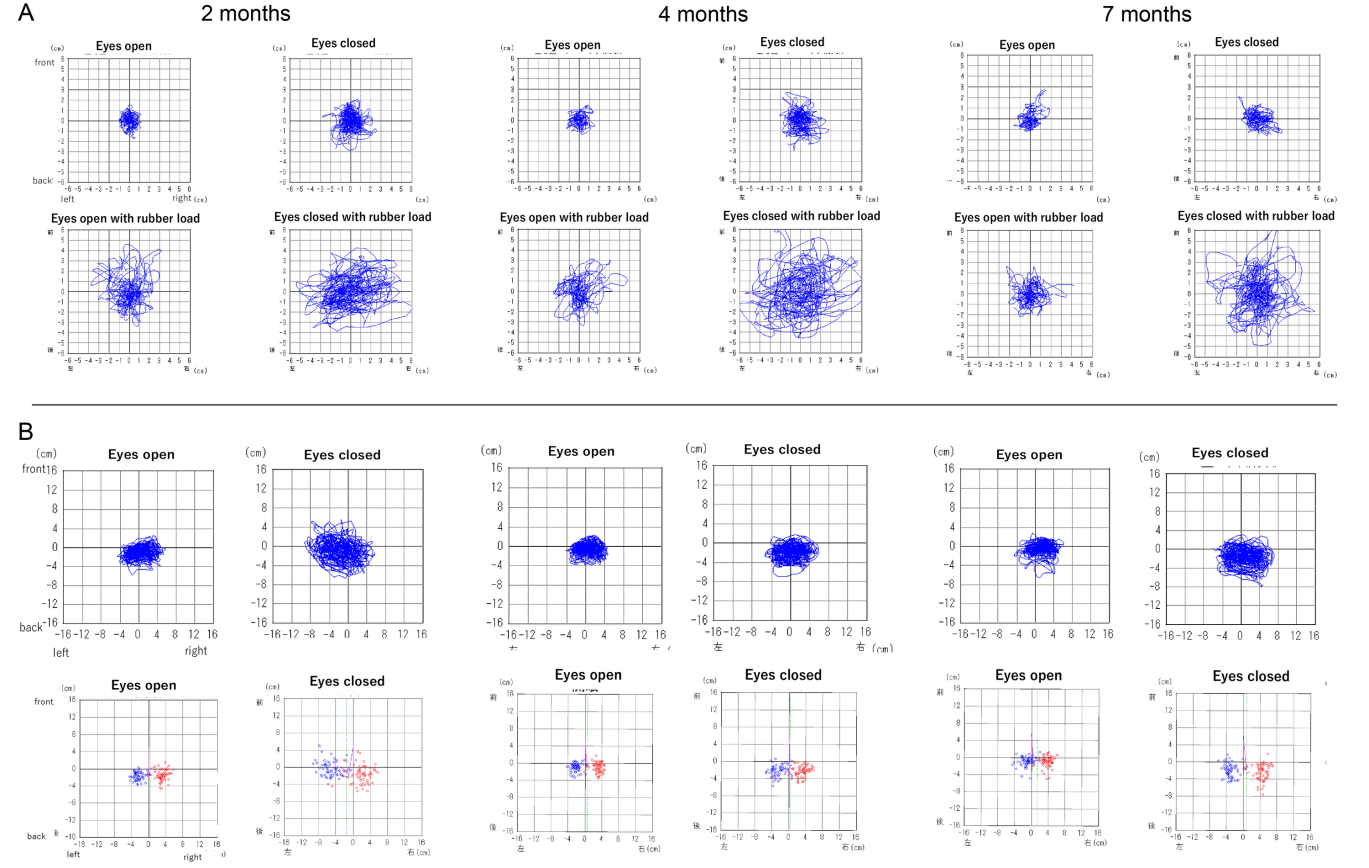
Course of posturography and Foulage test (A) Temporal changes in the center of pressure during static posturography. The four center of pressure diagrams for each condition are as follows: the upper left shows standing with eyes open; the upper right shows standing with eyes closed; the lower left shows standing with eyes open with a rubber load; and the lower right shows standing with eyes closed with a rubber load. The Y-axis represents front-back movement, and the X-axis represents left-right movement. Rubber loading reduces somatosensory input, allowing vestibular, visual, and somatosensory-dependent elements to be evaluated. (B) Temporal changes in the Foulage test. The four statokinesigrams for each condition are as follows: the upper left shows the center of pressure during stepping with eyes open; the upper right shows the center of pressure during stepping with eyes closed; the lower left shows the variances of steps with eyes open; and the lower right shows the variances of steps with eyes closed. Red indicates the distribution of the right foot’s center of pressure at the time of landing, and blue indicates the distribution of the left foot’s center of pressure. The Y-axis shows front-back movement, and the X-axis shows left-right movement. The Foulage test assesses dynamic equilibrium and determines susceptibility to disturbances.

Postural tests using a rubber load revealed instability with eyes closed and impaired dynamic balance in the Foulage test (Figures 2A, 2B). Head-up tilt (HUT) testing indicated instantaneous orthostatic hypotension (INOH) (Table 1). The patient was treated with antivertigo medication, adenosine triphosphate, and vitamin B12, along with vestibular rehabilitation exercises for gaze stabilization [16].

Follow-up

At four and seven months post-onset, the patient declined repeat caloric testing. By four months, oVEMP and Foulage test results showed slight improvement. At seven months, vHIT VOR gain improved (from 0.76 to 1.96), and oVEMP responses became nearly symmetrical (Figures 1B, 2B; Table 1), although values remained low, indicating a trend toward reduced responsiveness. DHI scores reflected reduced dizziness, and hospital anxiety and depression scale (HADS) scores were within normal range (Table 2). However, postural sway tests with rubber load indicated increased reliance on visual and proprioceptive input, and dynamic stability remained impaired (Figure 2A; Table 1). HUT testing suggested persistent autonomic dysfunction. Notably, left cVEMP p13 latency was prolonged from 14.6 ms to 26.1 ms (Figure 1A), suggesting sacculo-collic reflex impairment.

**Table 2:** Course of the questionnaire survey results DHI: dizziness handicap inventory, DHI-P: DHI physical, DHI-E: DHI emotional, DHI-F: DHI functional, HADS: hospital anxiety and depression scale, HADS-A: HADS anxiety, HADS-D: HADS depression.

Questionnaire	Subtype	Two months	Four months	Seven months	Reference (abnormal)
DHI	DHI-P	16	14	16	≧46
	DHI-E	4	6	2	
	DHI-F	14	10	4	
HADS	HADS-A	2	1	1	≧11
	HADS-D	4	3	2	

## Discussion

This case aligns with typical superior vestibular neuritis but presents potential COVID-19-related pathophysiological complications. The absence of cochlear involvement, unilateral vHIT gain reduction in the LSC, caloric hyporesponsiveness, and decreased oVEMP amplitudes indicate superior vestibular nerve dysfunction. However, elevated VZV antibodies suggest possible viral reactivation. Distinguishing between SARS-CoV-2-induced neural damage and secondary viral reactivation remains challenging.

Potential mechanisms include direct viral invasion, immune-mediated neuropathy, and vascular pathology. Viral reactivation in post-viral states has been linked to delayed vestibular symptoms, possibly triggered by COVID-19-related immune dysregulation, hypercoagulability, or endothelial dysfunction, leading to vestibular ischemia [17]. The immediate onset of dizziness post-infection in this case supports the direct viral insult hypothesis. Proposed entry routes of SARS-CoV-2 include transnasal propagation, hematogenous dissemination, and disruption of the blood-brain barrier, supported by postmortem and functional MRI showing brainstem alterations in patients with COVID-19 [17].

Recovery followed the expected trajectory for superior vestibular neuritis, with vHIT improvements over 6-12 months and oVEMP recovery lagging. However, persistent postural instability was a deviation from the norm. Postural sway tests indicated prolonged vestibular compensation, likely exacerbated by central processing deficits. Bilateral CP in caloric testing, prolonged cVEMP latency, and sustained orthostatic dysregulation in HUT testing suggested broader vestibulocochlear involvement beyond the superior vestibular nerve.

Table 3 summarizes case reports of vestibular neuritis associated with COVID-19 [7,8,11-17]. The ages of the patients ranged from 0 to 63 years, with a higher incidence in women. In only three cases did the onset of vertigo coincide with the detection of COVID-19 infection; in most cases, the onset varied from seven days to six weeks after infection, typically occurring relatively long after the initial infection. In most cases, IgG levels for herpes simplex virus (HSV) and VZV were not measured. Regarding vestibular function tests, most were not performed because of the severity of the vertigo symptoms, and only two case reports included quantitative tests. Of these, only one performed the same test after treatment. Among the cases where vertigo onset coincided with COVID-19 detection, we are the only ones to have performed quantitative balance function tests and observed the test results. To the best of our knowledge, this is the first report of its kind.

Furthermore, this case highlights long COVID-19-related neurological sequelae, including cognitive impairment (“brain fog”) and dysautonomia, which may contribute to prolonged convalescence [18]. Prolonged cVEMP latency suggests sacculo-collic reflex dysfunction, pointing to central involvement rather than purely peripheral pathology.

Although it remains unclear whether SARS-CoV-2 directly affected the superior vestibular nerve or triggered VZV reactivation, the atypical recovery, persistent autonomic dysfunction, and prolonged cVEMP latency indicate a broader impact on the vestibular and autonomic systems. Further research is needed to determine long-term SARS-CoV-2 effects on vestibular function and post-viral neurotropic infections.

This study has some limitations. First, as a single case report, the clinical course could only be evaluated based on changes in vestibular function test results, body balance assessments, and the patient's subjective symptoms, making statistical analysis unfeasible. Consequently, the definition of "recovery" remains ambiguous. Moreover, it is unclear whether the underlying cause of vestibular neuritis was the COVID-19 infection itself or a subsequent reactivation of VZV. To date, only one similar case has been reported in the literature [14], and further validation, potentially through animal studies, is warranted. Second, the disease course did not follow the typical trajectory of vestibular neuritis. The presence of symptoms resembling post-COVID-19 sequelae further complicated interpretation, limiting the ability to draw definitive conclusions. Nevertheless, this case report may provide useful insights should similar viral infections emerge in the future. Finally, the vHIT gain measured at seven months was 1.96, an unusually elevated value. Although technical or reproducibility issues may have contributed, the possibility of central overcompensation cannot be excluded [19]. Continued follow-up is planned, as the gain value may normalize over time.

**Table 3:** Literature review of COVID-19-related vestibular neuritis CP: canal paresis, vHIT: video head impulse test, ASC: anterior semicircular canal, LSC: lateral semicircular canal, cVEMP: cervical vestibular evoked myogenic potential, oVEMP: ocular VEMP.

Reference	Age	Sex	Time of vertigo onset (after COVID-19)	HSV or VZV IgG	Vestibular test	Follow-up vestibular test
Malayala and Raza (2020) [7]	20	F	0 days	None noted	Not implemented	None noted
Halalau et al. (2021) [8]	42	M	11 days	None noted	Not implemented	None noted
Aasfara et al. (2021) [9]	36	F	6 weeks	None noted	Caloric test: areflexia (CP)	Caloric test: complete recovery
Bokhary et al. (2021) [10]	23	F	10 days	None noted	HIT: positive. Gait: normal. Romberg sign: negative	None noted
Vanaparthy et al. (2020) [11]	63	F	4 weeks	Not implemented	None noted	None noted
Tannous and Klepper (2022) [12]	14	F	2 weeks	None noted	None noted	None noted
Bloomquist et al. (2023) [13]	9m	F	2 weeks	None noted	None noted	None noted
Mat et al. (2023) [14]	13	F	0 days	Positive	Fukuda stepping test; left deviation vHIT; left ASC and LSC decreased gain	None noted
Devaragudi and Gupta (2023) [15]	22	F	7 days	None noted	HIT: negative	None noted
Our case	57	M	0 days	Positive	Ref. Table 1	Ref. Table 1

## Conclusions

This case highlights a possible association between COVID-19 and vestibular neuritis, characterized not only by typical peripheral vestibular deficits but also by prolonged recovery and persistent balance dysfunction, raising the possibility of broader involvement, including the central nervous system. Although the clinical and diagnostic findings were compatible with superior vestibular neuritis, the atypical clinical course, marked by persistent postural instability, prolonged cVEMP latency, and orthostatic dysregulation, suggests that SARS-CoV-2 may exert additional effects on vestibular and autonomic pathways. The temporal proximity of vertigo onset to COVID-19 infection, along with gradual improvements observed in serial balance assessments, supports the hypothesis of COVID-19-related vestibular involvement. Importantly, this case offers rare longitudinal data from quantitative vestibular testing during recovery, highlighting the need for extended follow-up in similar patients to better characterize the potential spectrum of vestibular sequelae associated with COVID-19.
